# The effect of axial length on the short-term outcomes of cataract surgery combined with ab interno trabeculotomy

**DOI:** 10.1007/s00417-023-06337-1

**Published:** 2023-12-15

**Authors:** Hiroki Goto, Megumi Honjo, Takashi Omoto, Makoto Aihara

**Affiliations:** Department of Ophthalmology, University of Tokyo Graduate School of Medicine, 7-3-1 Hongo, Bunkyo-Ku, Tokyo, Japan

**Keywords:** Ab interno trabeculotomy, Glaucoma, Intraocular pressure, Axial length, Hyphema

## Abstract

**Purpose:**

Minimally invasive glaucoma surgery is safer and effective surgical modality for patients with glaucoma. To compare the effect of axial length (AL) on the surgical outcomes of combined cataract surgery and ab interno trabeculotomy (phaco-LOT), a retrospective, non-randomized comparative study was performed.

**Methods:**

In total, 458 eyes of 458 open-angle glaucoma patients who underwent phaco-LOT and were followed-up without any intervention for at least 6 months were enrolled. All were divided into a long-AL group (AL ≥ 26.0 mm, 123 eyes) and a not-long-AL group (AL < 26.0 mm, 335 eyes). The principal outcomes were the changes in intraocular pressure (IOP) and medication scores. We also sought a correlation between postoperative IOP spike and hyphema.

**Results:**

Significant postoperative reductions in IOP and medication scores were apparent in all subjects. The IOP reductions were significant at all timepoints in the not-long-AL group, but not until 1 month postoperatively in the long-AL group, and the IOP change was significantly lower in the long-AL group from postoperative day 1 to 3 months. On subanalysis of subjects by age, the microhook used, the pre-operative IOP, and the medication score, a significantly higher incidence of IOP spike was observed in the long-AL group in weeks 1 and 2 (both *p* < 0.05), but this did not correlate with hyphema status, implying that a different mechanism was in play.

**Conclusion:**

Phaco-LOT was effective regardless of AL, but the postoperative IOP decrease was lower and the early postoperative incidence of IOP spike was higher in long-AL eyes.

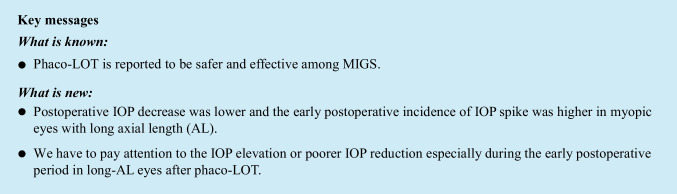

## Introduction

Lowering of intraocular pressure (IOP) is the gold standard treatment for glaucoma [1]. Trabeculotomy (LOT) cleaves the trabecular meshwork (TM) and inner wall of the Schlemm canal, which are the principal sites of resistance to aqueous outflow [2, 3]. Recently introduced minimally invasive glaucoma surgery (MIGS) is safer and less traumatic for patients with mild-to-moderate glaucoma that is uncontrolled by drops. Also, MIGS is valuable to reduce dependence on drops [4, 5]. Of the various MIGSs, several reports have suggested that procedures using microhooks such as the Kahook Dual Blade (KDB) or other hooks are simple, rapid, safe, and effectively reduce IOP. Ab interno trabeculotomy has become popular worldwide since last decade [6, 7].

Glaucoma prevalence increases with age, and cataracts often accompany glaucoma. Optimal management of both conditions depends on patient age, visual acuity, and glaucoma subtype and stage. When LOT is combined with cataract surgery (phaco-LOT), not only is the IOP lowered but also visual acuity improves. Phaco-LOT is often performed and effective in patients with primary open angle glaucoma (POAG), steroid-induced glaucoma, and exfoliation glaucoma (XFG) [8, 9].

Myopia is very common worldwide, and is a major risk factor for OAG [10–12]. Myopia is often accompanied by glaucoma. Many population-based studies have reported that OAG is associated with increased myopia, particularly in patients with moderate-to-high myopia. High myopia is an extreme form of myopia that accounts for 27–33% of all myopia, and is usually defined as a spherical equivalent less than − 6.00 diopters (D) or an axial length (AL) greater than 26.0–26.5 mm [13]. In patients with both myopia and glaucoma, combined cataract surgery and MIGS not only lowers IOP but also improves refractive error. At the same time, myopia is reported to be a risk factor for a postoperative IOP rise or IOP spike even after simple phacoemulsification [14, 15]. However, whether myopia affects surgical outcomes and IOP reduction remains unclear. Here, to evaluate the effects of AL on the outcomes of combined cataract surgery and phaco-LOT, postoperative IOPs and medication scores were compared between patients with long-AL and not-long-AL eyes. Postoperative hyphema is one of the most common complications after goniotomy procedures including LOT and can be associated with postoperative IOP increases [16, 17]. We thus assessed the association between the incidence of hyphema and IOP spike.

## Materials and methods

This study was approved by the institutional review board (IRB) of the University of Tokyo and was conducted in accordance with the principles of the Declaration of Helsinki. Informed consent was obtained from all patients before surgery.

### Subjects and data collection

This retrospective, observational comparative study included 458 consecutive eyes of 458 Japanese subjects (205 men, 253 women; mean age and standard deviation [SD] 70.6 ± 9.4 years). Data were retrieved for all subjects who fulfilled the inclusion criteria but not the exclusion criteria. All required surgical treatment and were using at least one antiglaucoma medication. The inclusion criteria included eyes that underwent phaco-LOT to control IOP and to treat visually relevant cataracts at the University of Tokyo Hospital between January 2018 and December 2021. The inclusion criteria were OAG including POAG, normal-tension glaucoma (NTG), XFG, steroid-induced glaucoma, and ocular hypertension; no history of intraocular surgery; and full postoperative evaluations on days 1 and 2, in weeks 1 and 2, and in months 1, 3, and 6. The exclusion criteria included any other contemporaneous surgery; any intraoperative complication, including posterior capsular rupture, Zinn zonular dialysis, or goniodialysis and any intervention within 6 months after surgery. If both eyes were eligible, the eye that underwent surgery earlier was chosen.

### Surgical procedure

Phaco-LOT via a 2.4-mm temporal corneal incision was accompanied by normal cataract surgery, including phacoemulsification and intraocular lens implantation. Two types of hooks, thus one of several spatula-shaped microhooks (M-2215S, 2215R, and 2215L, Inami, Tokyo, Japan) and a dual-blade hook (Kahook; KDB, New World Medical, Rancho Cucamonga, CA) were used. The choice of hook was at the discretion of the surgeon. A hook was inserted into the anterior chamber via the corneal port, and a Swan-Jacob gonioprism lens (Ocular Instruments, Bellevue, WA, USA) was then used to observe the opposite angle. A topical antibiotic (gatifloxacin) and a corticosteroid (0.1% [w/v] betamethasone sodium phosphate) were prescribed four times daily for 3–4 weeks postoperatively, and a miotic agent (pilocarpine hydrochloride) was prescribed three times daily. All drug doses were tapered as the postoperative course lengthened. IOP-lowering medications were re-commenced if the surgeon so decided.

### Statistical analysis

We recorded patient age, sex, glaucoma subtype, preoperative spherical equivalent refractive error (SERE), AL, pre- and post-operative IOPs, and number of antiglaucoma medications. All patients were divided into two subgroups: long-AL (AL > 26.0 mm) and not-long AL (AL ≤ 26.0 mm). The principal endpoints were the changes in IOP and medication scores over time. Observations were made on days 1 and 2, in weeks 1 and 2 (± 2 days), in months 1 and 3 (± 2 weeks), and in month 6 (± 4 weeks) postoperatively. IOP was measured by Goldmann applanation tonometry. The baseline IOP was the final IOP before surgery. The drug combination described above was coded “2”, and oral acetazolamide was coded “1” during medication-score analysis. Routine postoperative use of the miotic agent was not considered to lower the IOP. The IOP changes (%) and medication score ratio changes were obtained by dividing later scores by the changes between pre-operative and postoperative scores.

Sub-analyses were performed by matching the patient characteristics, thus age, the microhook chosen, and the pre-operative IOP and medication scores of patients who underwent phaco-LOT. An IOP spike was defined as an IOP elevation > 5 mmHg from baseline. Hyphema was defined as a niveau > 1 mm in height. Correlations between IOP spike and hyphema were sought at postoperative days 1 and 2, in weeks 1 and 2, and in month 1.

All continuous data are expressed as means ± SDs. The Fisher exact test and the *t* test were used (as appropriate) to compare patient demographics, postoperative complications, and reductions in IOP and medication scores. R version 3.4.3 (R Foundation for Statistical Computing, Vienna, Austria) was employed for all analyses. The level of statistical significance was set to *p* < 0.05.

## Results

### Patient demographics

**Fig. 1:**
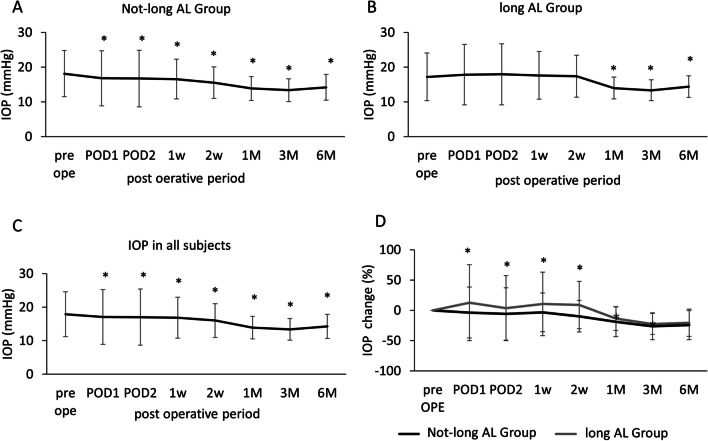
The IOP over time and comparison of the IOP change in all subjects. The intraocular pressures (IOPs) before surgery and at each follow-up for all, not-long-AL and long-AL subjects. **A** The IOPs of the not-long-AL group. **B** The IOPs of the long-AL group. **C** The IOP in all subjects. *p < 0.05 compared to the pre-operative values (*F* test). **D** The pre-operative IOP changes (%) and those after surgery. *p < 0.05 comparison between groups (*F* test)

**Fig. 2:**
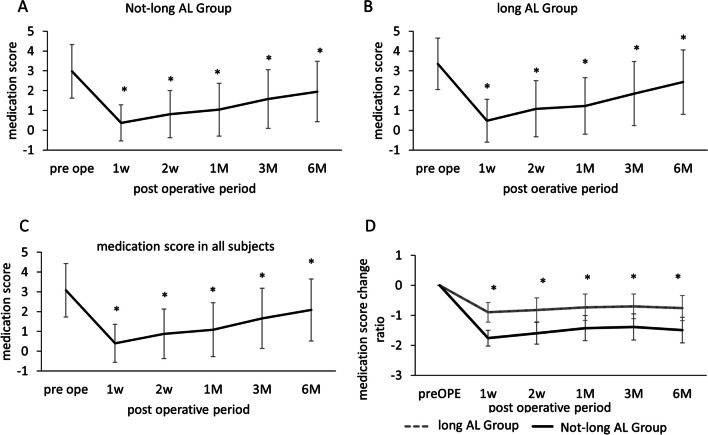
The medication score and comparison of the medicaiton score change ratio in all subjects. The medication score before surgery and at each follow-up for all, not-long-AL and long-AL subjects. **A** The medication scores of the not-long-AL group. **B** The medication scores of the long-AL group. **C** The medication scores in all subjects. *p < 0.05 compared to the pre-operative values (*F* test). **D** The medication score change raio and those after surgery. *p < 0.05 comparison between groups (*F* test)

In total, 463 eyes were included, and 5 eyes were excluded due to the additional trabeculectomy in 5 eyes; thus, 458 eyes were enrolled for analysis. Phaco-LOT used a microhook when treating 363 eyes and a KDB during surgery on 95 eyes. The average incisional angle was 98.8 ± 26.5°(range 60–240°). The average AL was 24.9 ± 2.0 mm (range 20.7–33.9 mm). The glaucoma subtypes were NTG (54 eyes), POAG (306), XFG (52), and secondary open-angle glaucoma (SOAG) (46). The average preoperative IOP and medication score were 17.9 ± 6.7 mmHg and 3.1 ± 1.4 respectively. All IOP and medication scores during the follow-up period are summarized in Figs. 1 and 2. The postoperative IOPs at 1, 3, and 6 months were 13.9 ± 3.4, 13.3 ± 3.2, and 14.2 ± 3.6 mmHg respectively. The postoperative medication scores at 1, 3, and 6 months were 1.1 ± 1.4, 1.7 ± 1.5, and 2.1 ± 1.0 respectively. For all patients, the IOP and medication scores were significantly lower than the pre-operative values at all postoperative timepoints; the p-values at 1, 3, and 6 months were < 0.0001, < 0.0001, and < 0.001, respectively) (Figs. 1C and 2C). The type of hook used did not affect these values at 6 months.

### Surgical outcomes of the not-long and long-AL groups

Next, we divided all patients into two groups by the AL; patient demographics are summarized in Table 1. Sex, AL, age, hook used, and pre-operative medication score all differed significantly between the groups, but eye laterality, glaucoma subtypes, and pre-operative IOP did not.

**Table 1:** Patient demographics and characteristics (all subjects)

	Not-long-AL group	Long-AL group	*P* value
Sex	127/208	78/45	< 0.0001
AL (mm)	24.0 ± 1.1	27.6 ± 1.6	< 0.0001
AL (mm)	24.0 ± 1.1	27.6 ± 1.6	< 0.0001
Glaucoma subtype	490/221/40/34	15/84/12/12	N.S
NTG/POAG/XFG/SOAG			
Hook (KDB/micro)	78/257	17/106	0.03
Baseline IOP (mmHg)	18.1 ± 6.6	17.1 ± 6.7	N.S
Baseline medication score	3.0 ± 1.4	3.3 ± 1.3	< 0.05

Data are shown as means ± standard deviations. *AL* Axial length, IOP: intraocular pressure, *NTG* normal tension glaucoma, *POAG* primary open-angle glaucoma, *XFG* exfoliation glaucoma, *SOAG* secondary open-angle glaucoma; *t* test for age, AL, preoperative baseline IOP, and medication score. *F* test for sex, eye laterality, glaucoma subtypes, and the hook used

The changes in IOP during follow-up are shown in Fig. 1. In the not-long-AL group, a significant IOP decrease from baseline was observed at all follow-up timepoints, but, in the long-AL group, the fall was not significant until 1 month postoperatively (Fig. 1A, B). The IOPs and medication scores at 6 months were 14.37 ± 3.10 and 14.18 ± 3.73 mmHg, and 2.43 ± 0.81 and 1.95 ± 0.42, in the long and not-long-AL groups respectively. Although there was no significant difference in the 6-month IOPs, the postoperative IOP change was significantly less in the long-AL group from 1 day to 3 months postoperatively; the *p* values at 1 and 2 weeks and 1 and 2 months were < 0.01, = 0.01, < 0.01, < 0.01, = 0.01, and = 0.02 respectively, as shown in Fig. 1D.

The pre- and post-operative medication scores in not-long and long-AL groups are shown in Fig. 2A and B. These were significantly lower than the pre-operative scores in both groups. However, as indicated in Fig. 2D, the change was significantly less in the long-AL group than in the not-long-AL group during follow-up. Although the pre-operative medication scores differed between the groups, it is possible that IOP lowering was less effective in the long-AL group than in the not-long-AL group in the early postoperative period.

### Sub-analyses of the surgical outcomes of the not-long and long-AL groups

**Fig. 3:**
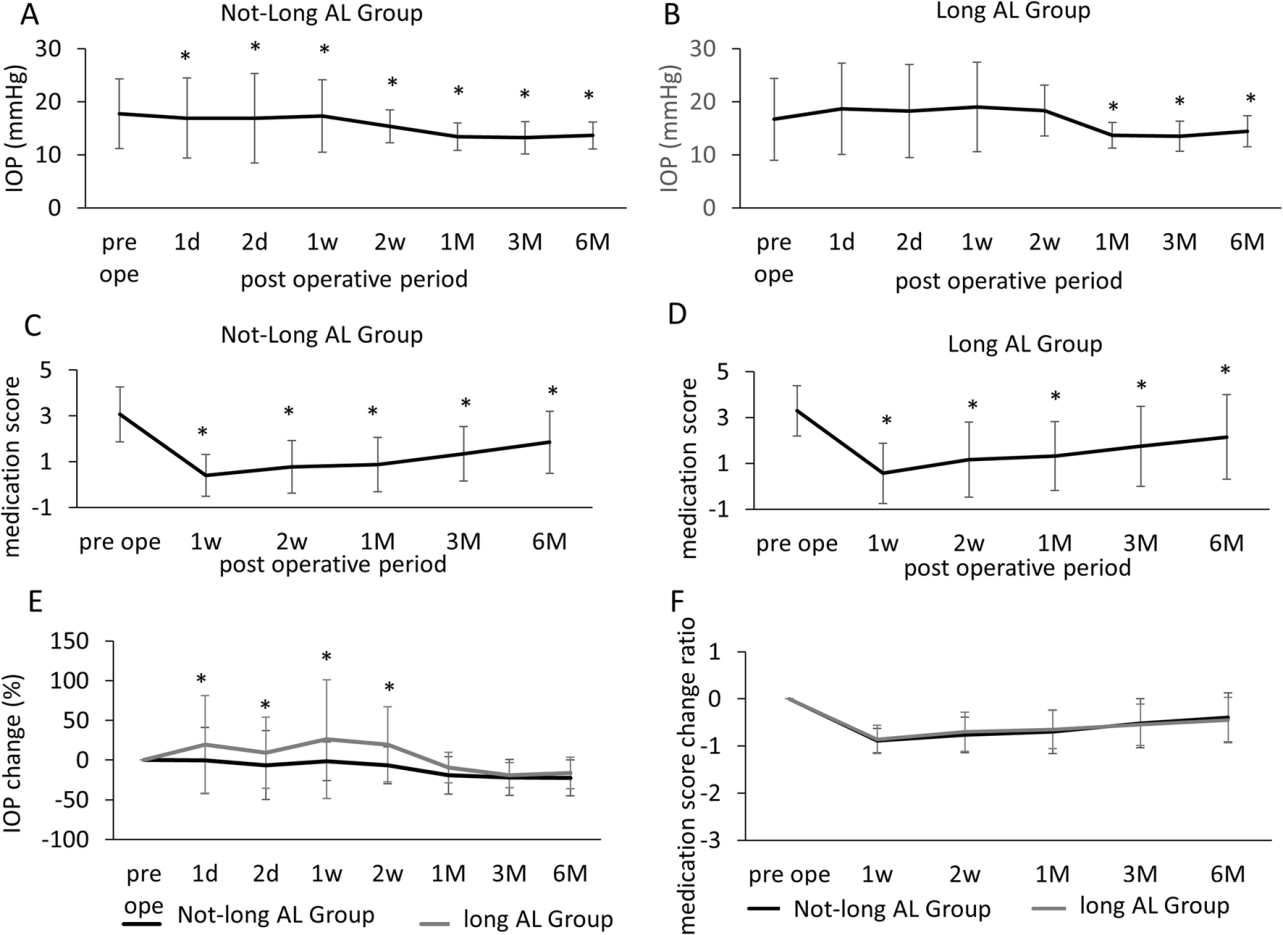
The IOP and medication scores change over time in subanalysis. The IOP and medication scores before surgery and at each follow-up of the not-long AL and long-AL in subanalysis. **A** The IOPs of the not-long-AL subgroups. **B** The IOPs of the long-AL subgroups. **C** Medication scores of the not-long-AL subgroups. **D** Medication scores of the long-AL subgroups. *p < 0.05 compared to the pre-operative medication scores (F test). The differences in the **E** IOPs (%) and **F** medication score change ratio between the not-long-AL and long-AL subgroups before surgery and at each follow-up in subanalysis. *p < 0.05 (*F* test)

We next performed subanalyses of the effects of age, hook choice, pre-operative IOP, and medication scores on the outcomes. The subgroup demographics are shown in Table 2. The time courses of IOP and medication scores are shown in Fig. 3. As was true overall, postoperative IOPs and medication scores fell significantly in all subgroups. The changes in medication scores were similar in all subgroups, but the IOP fell significantly less in the long-AL subgroups than in the not-long-AL subgroups at 1 and 2 days and 1 and 2 weeks (thus before 1 month) postoperatively (Fig. 3B). The absence of any significant differences in the medication score changes is attributable to the fact that the pre-operative medication score was adjusted prior to all subanalyses. However, such subanalyses implied that IOP lowering during the early postoperative period was poorer in the long-AL subgroups than in the not-long-AL subgroups.

**Table 2:** Patient characteristics and the group frequencies of IOP spike and hyphema in subanalysis

	Not-long AL (*n* = 44)	Long AL (*n* = 44)	*P* value
AL (mm)	24.3 ± 1.2	27.7 ± 1.7	< 0.01
Age (years)	68.3 ± 6.5	67.7 ± 7.4	0.24
Glaucoma subtype NTG/POAG/XFG/SOAG	6/28/10/0	7/30/7/0	N.S
Preoperative IOP (mmHg)	17.7 ± 6.5	16.7 ± 7.7	0.08
Preoperative medication score	3.07 ± 1.19	3.30 ± 1.10	0.32
Hyphema (postoperative time)			
1 day	4	9	0.13
1 week	1	4	0.17
2 weeks	1	0	0.31
1 month	1	0	0.31
IOP spike (postoperative time)			
1 day	8	12	0.31
1 week	5	14	0.02
2 weeks	1	7	0.03
1 month	1	0	0.31

*t* test for AL, age, and the preoperative baseline IOP, medication scores, hyphema, and IOP spike

We next sought correlations between IOP spike and hyphema in each subgroup; these are thought to be related (Table 2). There was no significant among-subgroup difference in hyphema status, but IOP spike was significantly more common in long-AL patients than in not-long-AL patients in weeks 1 and 2. However, no correlation was apparent between the incidence of hyphema and IOP spike (*p* = 0.1453–0.5813, *F* test). Thus, postoperative IOP spike to 1 month, which was more common in the long-AL group than in the not-long-AL group, may not be associated with hyphema.

## Discussion

Phaco-LOT safely and significantly reduced IOP and medication scores in both myopic and non-myopic eyes. There was no significant difference in either the postoperative IOP or medication score at 6 months among all subjects, but the IOP fall was slower to 1 month postoperatively in the long-AL group. We performed subanalyses of the effects of age, hook choice, preoperative IOP, and medication scores on the outcomes. A significantly higher incidence of IOP spike was apparent in the long-AL group than in the not-long-AL group in weeks 1 and 2 postoperatively, but this did not correlate with hyphema status; IOL spike seems to be attributable to some other mechanism. Thus, phaco-LOT usefully treats highly myopic eyes, although the IOP decrease was less and the incidence of IOP spike was higher in long-AL eyes than in not-long-AL eyes over the early postoperative period.

Only a few previous reports have focused on the effectiveness of LOT or phaco-LOT for patients with myopic glaucoma; most studies enrolled both myopic and non-myopic eyes [5–7, 16, 17]. Recently, Yoshida et al. compared the surgical results of LOT alone using a Kahook Dual Blade. Patients with 20 high-myopic eyes and 20 non-high-myopic eyes were enrolled; the postoperative success rate at 36 months was significantly lower (45%) for highly myopic eyes than for non-highly myopic (65%) eyes [18]. Pathological myopia was a significant risk factor for surgical failure in highly myopic eyes. The cited authors found, as did we, that the IOP decrease was inferior in long-AL eyes compared to that in not-long-AL eyes in the early postoperative period.

Although the mechanisms remain unclear, early transient IOP spike or a persistently high IOP is a common, and possibly serious, adverse event after LOT or phaco-LOT. Visual-field defects may progress or an additional surgical procedure such as filtration surgery may be needed if a high IOP persists after surgery, especially in patients with advanced glaucoma. Several studies have suggested that, in patients with high myopia, the aqueous outflow through the trabecular meshwork may be reduced in those with long-AL eyes; the network thickness and the vertical diameter of the Schlemm canal are smaller in eyes with high myopia than in non-myopic eyes [19], which is attributable to AL elongation and metabolic dysfunction in long-AL eyes. Thus, it has been suggested that long-AL status is a risk factor for early IOP elevation after cataract surgery on highly myopic eyes [19, 20].

In general, IOP-lowering effects in glaucoma and non-glaucoma patients, even after cataract surgery alone, have long been known [21]. Thus, during phaco-LOT, the combined procedure may enhance the IOP-lowering effect of cataract surgery alone; the angle is widened and aqueous outflow enhanced [22]. However, one study reported that IOP-lowering was less marked in long-AL eyes than in not-long-AL eyes after cataract surgery; the IOP changes after such surgery and the relationships thereof with refractive conditions were investigated [23]. Significant IOP reductions were observed early postoperatively (thus at days 7 and 30) in patients with emmetropic and mild-to-moderate myopia. However, for those with high myopia, the IOP was unstable from 1 to 30 days and lower only at 90 days. These results are similar to ours; the IOP of highly myopic patients fell slowly and was unstable during the first postoperative month, although we performed phaco-LOT.

Phaco-LOT is associated with fewer complications than other glaucoma surgeries such as filtration surgery [24]. However, usually, medications are required to control the postoperative IOP. IOP spikes and IOP increases have been reported in up to 20% of patients [25], and hyphema in 4.6–33.9%; the latter is thought to be a major risk factor for IOP spike [26–28]. The blood reflux is a valuable indicator of correct trabecular pathway incision and aqueous outflow enhancement, but the IOP occasionally increases given the associated hyphema. However, in our present study, the incidence of IOP spike was higher in the long-AL group than in the not-long-AL group, but was not associated with hyphema; another mechanism is in play.

Early, transient IOP spikes are common postoperative complications after all intraocular surgeries [29], but such IOP spikes are more frequent in highly myopic eyes than in non-myopic eyes even after simple cataract surgery, possibly attributable to the abovementioned differences in the anatomical and pathological features of the trabecular pathway in long-AL eyes. Apart from low aqueous outflow, several other explanations of the higher incidence of postoperative, early, transient IOP spikes in highly myopic eyes have been suggested. First, such myopic eyes are at greater risk of blood-aqueous barrier (BAB) breakdown than are non-myopic eyes. One previous study reported elevated inflammatory cytokine levels and prolonged anterior chamber cell reactions that persisted for 3 weeks after cataract surgery in highly myopic eyes [30]. Second, highly myopic eyes have larger anterior segments, and may thus be more prone than other eyes to blockage of the TM by viscoelastic material that is not completely removed after surgery. Lastly, long-AL eyes are less rigid than not-long-AL eyes, perhaps rendering the former eyes vulnerable to surgical trauma that triggers prolonged inflammation [31]. As we combined two procedures, the IOP elevations may have been caused by that combination. Anterior segment optic coherence tomography (AS-OCT) should be used to further explore the causes of IOP spike after phaco-LOT in long-AL eyes, which differ in terms of structure and function from not-long-AL eyes.

Postoperative steroids sometimes elevate IOP. Topical corticosteroids increased the incidence of IOP spikes within 3 months after gonioscopy-assisted transluminal trabeculotomy [32]. In another report, the incidence of postoperative steroid responders was relatively low, but younger age and a higher AL were risk factors for a steroid response after cataract surgery [33]. The risk of postoperative IOP elevation after phaco-LOT and the relationship thereof with AL warrants further examination.

The limitations of this study include its retrospective nature, the lack of sample randomization, and the fact that the surgical procedures varied (the surgeons, hooks, and trabeculotomy ranges differed). Also, we lack long-term follow-up data. Although there were no significant differences between the number of excluded eyes (4 eyes in the not-long-AL eyes and 1 eye in the long-AL eyes) within the follow-up period in the present study, further prospective study with longer follow up period would provide valuable insights into the long-term effectiveness and safety of phaco-LOT. In addition, a comparative study with the group undergoing a different minimally invasive glaucoma surgery would enable a more comprehensive evaluation of treatment effectiveness [34, 35]. However, we show that certain factors influence IOP elevation or poorer IOP reduction in long-AL eyes after phaco-LOT, especially in the early postoperative period. A long-term prospective analysis is needed to identify additional factors associated with the surgical outcomes of long-AL eyes.
